# Medical management of first trimester missed miscarriages - A cross-sectional study

**DOI:** 10.12669/pjms.40.7.8751

**Published:** 2024-08

**Authors:** Shatha Nasser, Tazeen Makhdoom, Laila Yahya Ahmad Alhubaishi, Hassan M Elbiss

**Affiliations:** 1 Shatha Nasser, MBBS. Department of Obstetrics and Gynecology, Latifa Hospital, Dubai, United Arab Emirates; 2 Tazeen Makhdoom, MBBS. Department of Obstetrics and Gynecology Latifa Hospital, Dubai, United Arab Emirates; 3 Laila Yahya Ahmad Alhubaishi, MBBS. Department of Obstetrics and Gynecology, Latifa Hospital, Dubai, United Arab Emirates; 4 Hassan M Elbiss, MD, CCT, FRCOG. Department of Obstetrics and Gynecology, College of Medicine and Health Sciences, United Arab Emirates University, Al-Ain, United Arab Emirates

**Keywords:** First trimester miscarriage, Missed miscarriage management, Misoprostol

## Abstract

**Background & Objective:**

Miscarriage, a common complication of early pregnancy before 12 completed weeks of gestation, is typically managed medically. We aimed to estimate the success and complication rate of medical management in women with first-trimester missed miscarriages. Our objective was to calculate the rate of complete uterine evacuation within three weeks of treatment, rate of infection, significant blood loss, re-admission, or surgical evacuation.

**Methods:**

It was a retrospective cross-sectional study that included women diagnosed with miscarriage at less than 13 weeks’ gestation in Latifa Hospital’s Gynecology Department from January 2019 to December 2019 in Dubai. These patients were given vaginal misoprostol, 400-800 mcg every 6-8 hours until expulsion of pregnancy.

**Results:**

There were 294 women included in the study. The success rate was 60.5% (178/294). Twenty women developed significant blood loss (6.8%), four women developed infection (1.4%), 76 required readmission (25.9%), 12 women received blood transfusion (4.1%), and 74 women required a surgical evacuation (25.2%). Nulliparity, unscarred uterus, and the presence of abdominal pain with vaginal bleeding before treatment were significantly associated with the successful medical treatment (p<0.05).

**Conclusion:**

The success rate of the medical regimen studied lies on the lower end of what is quoted in the literature. The difference in the success rate could be attributed to the different definitions of success in other studies. Nulliparity, unscarred uterus and presence of abdominal pain with vaginal bleeding were associated with higher success.

## INTRODUCTION

Miscarriage is one of the commonest early pregnancy complications encountered in clinical practice.^1^-^3^ Majority of miscarriages occur before 12 completed weeks of gestation.^4^ While surgical treatment of miscarriage is highly effective, it is an invasive procedure that carries risks. Possible short-term complications of dilatation and curettage are uterine perforation, cervical trauma, and anesthesia-related complications amongst others.^5^ An important long-term complication is intrauterine adhesions (IUAs) and the consequences on reproductive performance.^6^,^7^ Medical management of miscarriage has been increasingly in use, providing an alternative to surgery and avoiding the risks and costs of surgery and anesthesia.^8^,^9^ Medical management, consisting of misoprostol administered orally, sublingually, or vaginally with or without prior priming with oral mifepristone, is reported to provide a greater success rate compared to expectant management with a comparatively similar complication rate.^4^,^10^-^14^

Success rates of medical management of missed miscarriages range somewhere between 60-90%.^4^,^8^,^11^,^12^,^15^,^16^ A 2022 randomized controlled trial (RCT) comparing medical management of missed miscarriage by mifepristone and misoprostol versus misoprostol alone found that mifepristone plus misoprostol was more effective than misoprostol alone.^12^ There was no difference in incidence of adverse events in both groups. Another RCT that compared efficacy of vaginal and sublingual misoprostol for managing missed miscarriages found no significant difference between two routes.^15^ However, sublingual misoprostol was associated with an unpleasant taste which is not well tolerated by the patients.

Positive results of medical management of missed miscarriages were found in a double-blinded placebo-controlled study where 64 miscarriage patients were randomized to a single dose of vaginal misoprostol 400 mcg and another 62 patients to placebo (success rates of 81% and 52% respectively were achieved within one week).^16^ Medical management of miscarriage may provide an alternative to surgical evacuation, but there is no consensus on the optimum misoprostol regimen.^11^ This makes it difficult when counselling women about their treatment options. The objective of this study was to measure the success and complication rates of a specific misoprostol regime in women with miscarriage.

## METHODS

It was a retrospective cross-sectional study.The records of women diagnosed with miscarriage at the early pregnancy unit and gynecology ward at Latifa Hospital in Dubai were reviewed for the entire 2019 year.

### Ethical Approval:

It was approved by the Dubai Scientific Research Ethics Committee (Ref no: DSREC/RRP/2021/20). Date September 13, 2022.

Women who were diagnosed with missed miscarriage at a gestational age of less than 13 weeks and received vaginal misoprostol as the primary treatment were included in the study. Women who were diagnosed with miscarriage at a gestational age of more than 13 weeks or with incomplete miscarriage, underwent surgical evacuation as the primary treatment, and those who failed to attend the follow-up appointment were excluded from the study. The diagnosis of missed miscarriage was based on an ultrasound finding of an absent fetal heart activity when the crown-rump length measured more than 6 mm or an absent fetal pole when the gestational sac measured more than 25 mm. Patients were treated with 400-800 mcg of misoprostol administered vaginally every six to eight hours until abortion occurred, according to the local pharmacy protocol. A maximum of three doses were given. Patients were given a follow up appointment within three weeks on average after discharge from the hospital which consisted of a pelvic ultrasound or a pregnancy test or both.

Among all patients diagnosed with first trimester missed miscarriage and managed medically, the number of cases with successful treatment, as well as the number of cases with significant blood loss, infection after starting misoprostol, readmission, and surgical evacuation were recorded. Clinical variables consisting of parity index, presence of a uterine scar, presence of a fetal pole on the pre-treatment ultrasound, gestational age at diagnosis, and pre-treatment symptoms (including those who presented without any symptoms described in textbook terms as missed abortion) were also recorded with the intent of assessing their association with the success of the treatment.

Successful treatment was defined as complete uterine evacuation or incomplete uterine evacuation, which was successfully managed expectantly on the lines of published guidelines^17^ without readmission or need for surgical evacuation. Complete uterine evacuation was defined as an ultrasound finding of an anteroposterior endometrial diameter of =/<15 mm, or a negative pregnancy test if an ultrasound was not performed and the woman was asymptomatic of bleeding. Significant blood loss was defined as a drop in hemoglobin by more than 2g or bleeding that necessitated blood transfusion or emergency evacuation. Infection was defined as the presence of purulent vaginal discharge, fever of 38 degrees Celsius or more, tender uterus, and high inflammatory mark.

### Statistical Analysis:

Data analysis was done using the Statistical Package for Social Sciences (SPSS) version 21. Continuous variables with normal distribution were presented as means and standard deviations, and non-parametric continuous variables as medians and interquartile range. Categorical variables were given as numbers (percent) and associations were tested using the Pearson Chi-square test. Differences were regarded as statistically significant if the p*-*value was less than 0.05.

## RESULTS

The records of 379 women diagnosed with miscarriage were reviewed. Two hundred and ninety-four women met the inclusion criteria. The mean age of included patients was 33.4 years (range 17-45 years), Out of 294 women, 234 (79.6%) had missed miscarriage and 60 (20.4%) had anembryonic pregnancy. Seventy-four (25.2%) women were nulliparous, 124 (42.2%) women had one or two previous births, 80 (27.2%) women had three to five previous births, and 16 (5.4%) women had more than five previous births (Table-I). One hundred and eighty-six (63%) women were diagnosed at seven weeks or less and 108 (36%) women were diagnosed between seven and 13 weeks.

**Table-I:** Characteristics of study sample.

Characteristics	No (%) (n=294)
***Age group (years)***	
17-25	41 (13.9)
26-35	121 (41.2)
36-40	90 (30.6)
41-45	42 (14.3)
***Parity Index***	
P0	74 (25.2)
P1-2	124 (42.2)
P3-5	80 (27.2)
>P5	16 (5.4)
***Gestational age (weeks)***	
≤ 7	186 (63.3)
7-13	108 (36.7)
***Uterine scar***	
Present	122 (41.5)
Absent	172 (58.5)
***Fetal pole***	
Present	234 (79.6)
Absent	60 (20.4)
***Uterine scar***	
Present	122 (41.5)
Absent	172 (58.5)
***Symptoms***	
Asymptomatic	132 (44.9)
Abdominal pain	26 (8.8)
Per vaginal bleeding	58 (19.7)
Abdominal pain and per vaginal bleeding	78 (26.3)

The success rate of medical management was 60.8% (Table-II). Gestational age of less than seven weeks was associated with higher success rate (64.52%) as compared to gestational age between seven and 13 weeks (53.77%), an association that was not statistically significant (p=0.067) (Table-III). Nulliparity and unscarred uterus were strongly associated with the highest success rate (p<0.01) (Table-III). The previous three to five births were associated with the lowest success rate (p <0.01) (Table-III). Presence of both abdominal pain and vaginal bleeding on presentation was associated with increased success rate of medical management. (p <0.01) (Table-III).

**Table-II:** Medical treatment of first trimester missed miscarriage outcome.

Outcome	No (%)
Successful treatment	
Complete evacuation	160 (34.42)
Incomplete evacuation managed expectantly	16 (5.44)
Asymptomatic patient with negative pregnancy test after 3 weeks	3 (1.02)
Total	179 (60.88)
Failed treatment	
Complete evacuation after Surgical evacuation done	74 (24.83)
Complete evacuation after second trial of medical management	42 (14.29)
Total	115 (39.12)

**Table-III:** Association between treatment outcome and gestational age, parity, uterine scarring and symptoms.

	Successful treatment	Failed treatment	p-value
***Gestational Age***			
>7 weeks	120	66	0.067
7-13 weeks	58	50	
***Parity***			
P0	57	17	<0.01
P1-2	74	50	
P3-5	38	42	
>P5	10	6	
***Uterine scar***			
Present	52	70	<0.01
Absent	127	45	
***Symptoms***			
Asymptomatic	72	60	<0.01
Abdominal pain	10	16	
Per vaginal bleeding	32	26	
Abdominal pain and per vaginal bleeding	65	13	

**Fig.1:**
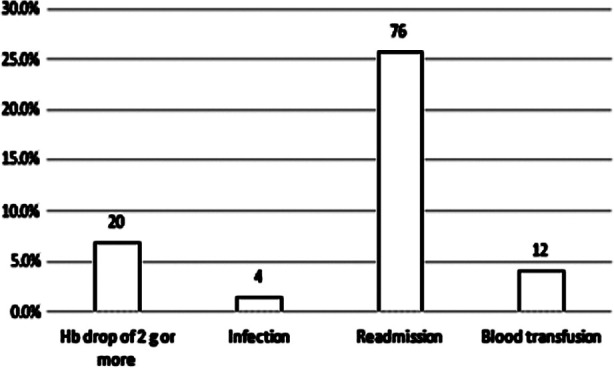
Complications in medical management of first trimester missed miscarriage.

The overall complications that were encountered in these patients were; hemoglobin drop of more than two grams, infection, blood transfusion, and readmission. Four (1.4%) women developed infection, 76 (25.9%) required readmission, 12 (4.1%) women received blood transfusion, and 73 (24.8%) required a surgical evacuation. (Fig.1).

## DISCUSSION

This study found that the misoprostol regimen used for first trimester missed miscarriage had a successful result in more than half of the treated women. Women who were nulliparous, had unscarred uterus, or were symptomatic at the time of presentation responded well to the medical treatment with misoprostol.

Our findings should be interpreted in context with prior published studies. A retrospective cohort study including 296 women, used a single dose of 800 mcg of vaginal misoprostol and showed a success rate of 67%, even though it included patients of incomplete miscarriage as well.^18^ Our study showed similar success rates i.e. 60.2%. However, we included only those patients who had first trimester miscarriage. A randomized controlled trial (RCT) conducted in Pakistan compared the efficacy of manual vacuum aspiration (MVA) and misoprostol in management of first trimester incomplete miscarriage.^19^ Both treatments had higher efficacy rate i.e. >90% but MVA group had more success rate when compared to the misoprostol group. In our cohort study, we only focused on misoprostol regime and found comparatively lower success rates.

Another experimental study showed effective results of misoprostol regime for managing missed miscarriages (either via sublingual or vaginal route)^15^ Sublingual route had a drawback of unpleasant taste and adverse effects were seen in 72% of the women treated via sublingual route. Our study only focused on the vaginal route for administering misoprostol. An RCT was conducted in Skåne University Hospital, Sweden and included women with early non-viable pregnancy and vaginal bleeding.^20^ Vaginal misoprostol treatment resulted in complete uterine evacuation in 66% of the patients. Our study also showed comparable success rates i.e. 60.8%. In general, the results across literature provide strong evidence in favor of medical management of first trimester missed miscarriages. There exist some differences in success rate of various studies owing to differences in dose, route of administration, inclusion criteria and definition of success.

The study was reported according to the guidelines mentioned in the STROBE checklist.^21^ This was a retrospective study that depended on records review. Thus, significant bleeding that required surgical evacuation could not be defined in the study. Another limitation of our study was that we used a range of dose and did not address the question of exact dose finding. An objective measure of the bleeding was not provided consistently in the records. One reason why this study showed a lower success rate than other similar studies which used 26 multiple doses of misoprostol could be that it included exclusively missed miscarriages while other studies included incomplete miscarriages as well.

In view of the increasing rate of cesarean delivery, a future study to elucidate the relationship between uterine scar and treatment failure would be very helpful in patient’s counseling. Such study would be most helpful in forming recommendations if it compares the rates of treatment failure, significant bleeding, and uterine rupture between patients with uterine scar and without uterine scar who received the same regimen of misoprostol.

## CONCLUSION

The medical management of first trimester missed miscarriages can avoid surgical evacuation and related complications. Patients opting for this method might require hospital readmission for further treatment in case of failure. Our study provides quantified estimations of the success and complication rates to counsel women in an evidence-based manner.

### Authors’ contributions:

**SN:** Conceived, designed, did the statistical analysis, data collection. Responsible and accountable for the accuracy and integrity of the work.

**TM:** Assisted in manuscript writing, analysis and data collection.

**LYAA:** Assisted in data collection, manuscript editing and formatting.

**HME:** Assisted in the data analysis, manuscript writing, editing and formatting. Responsible and accountable for the accuracy or integrity of work.
